# Does Cathodal vs. Sham Transcranial Direct Current Stimulation Over Contralesional Motor Cortex Enhance Upper Limb Motor Recovery Post-stroke? A Systematic Review and Meta-analysis

**DOI:** 10.3389/fneur.2021.626021

**Published:** 2021-04-15

**Authors:** Joyce L. Chen, Ashley Schipani, Clarissa Pedrini Schuch, Henry Lam, Walter Swardfager, Alexander Thiel, Jodi D. Edwards

**Affiliations:** ^1^Heart and Stroke Foundation Canadian Partnership for Stroke Recovery, Sunnybrook Research Institute, Toronto, ON, Canada; ^2^Faculty of Kinesiology and Physical Education, University of Toronto, Toronto, ON, Canada; ^3^Rehabilitation Sciences Institute, University of Toronto, Toronto, ON, Canada; ^4^Sunnybrook Health Sciences Centre, Toronto, ON, Canada; ^5^Department of Pharmacology, University of Toronto, Toronto, ON, Canada; ^6^Lady Davis Institute, Jewish General Hospital, Montreal, QC, Canada; ^7^Department of Neurology and Neurosurgery, McGill University, Montreal, QC, Canada

**Keywords:** stroke, transcranial direct current stimulation, upper limb, motor recovery, systematic review and meta-analysis

## Abstract

**Background:** During recovery from stroke, the contralesional motor cortex (M1) may undergo maladaptive changes that contribute to impaired interhemispheric inhibition (IHI). Transcranial direct current stimulation (tDCS) with the cathode over contralesional M1 may inhibit this maladaptive plasticity, normalize IHI, and enhance motor recovery.

**Objective:** The objective of this systematic review and meta-analysis was to evaluate available evidence to determine whether cathodal tDCS on contralesional M1 enhances motor re-learning or recovery post-stroke more than sham tDCS.

**Methods:** We searched OVID Medline, Embase, and the Cochrane Central Register of Controlled Trials for participants with stroke (>1 week post-onset) with motor impairment and who received cathodal or sham tDCS to contralesional M1 for one or more sessions. The outcomes included a change in any clinically validated assessment of physical function, activity, or participation, or a change in a movement performance variable (*e.g*., time, accuracy). A meta-analysis was performed by pooling five randomized controlled trials (RCTs) and comparing the change in Fugl–Meyer upper extremity scores between cathodal and sham tDCS groups.

**Results:** Eleven studies met the inclusion criteria. Qualitatively, four out of five cross-over design studies and three out of six RCTs reported a significant effect of cathodal vs. sham tDCS. In the quantitative synthesis, cathodal tDCS (*n* = 65) did not significantly reduce motor impairment compared to sham tDCS (*n* = 67; standardized mean difference = 0.33, *z* = 1.79, *p* = 0.07) with a little observed heterogeneity (*I*^2^ = 5%).

**Conclusions:** The effects of cathodal tDCS to contralesional M1 on motor recovery are small and consistent. There may be sub-populations that may respond to this approach; however, further research with larger cohorts is required.

## Introduction

More than 60% of stroke survivors have persistent motor deficits for months to years after stroke (1). Innovative approaches to rehabilitation that improve motor recovery are required to reduce the burden of disability post-stroke. Transcranial direct current stimulation (tDCS) is a type of neuromodulatory non-invasive brain stimulation that shifts cortical excitability into a relative state of inhibition or excitation. The pairing of upper extremity rehabilitation therapies with tDCS has potential to facilitate recovery beyond that achieved with rehabilitation alone (2, 3). Many tDCS applications in stroke are based on a model of interhemispheric inhibition (IHI) (4, 5) thought to regulate cortical excitability between left and right motor cortex *via* transcallosal fibers. Specifically, the IHI model postulates that recovery is hindered because of reduced inhibition from ipsilesional motor cortex (M1) to contralesional M1. This leads to “over-active” contralesional networks that are thought to be less efficient or to even inhibit recruitment of damaged ipsilesional networks. The inhibition of contralesional M1 using tDCS with the cathode over this region may down-regulate the over-activity and promote increased functional recovery (5). Therefore, we performed a systematic review and meta-analysis to determine whether the existing evidence indicates that cathodal tDCS to contralesional M1 enhances motor performance and/or recovery after stroke.

Many studies apply cathodal tDCS to contralesional M1 with the rationale to rebalance impaired interhemispheric interactions and restore function (6). Contralesional neural activity is associated with poor motor outcome (7–13), and IHI is impaired post-stroke (14–16). However, recent discussions have questioned the validity of the impaired IHI model (17). Some studies report no evidence of over-activation in contralesional M1 or impairment in IHI post-stroke (18, 19). A one-size-fits-all model for the use of tDCS to treat stroke motor dysfunction may be insufficient (4, 18–22). For example, individuals with severe motor impairment do not improve with cathodal tDCS to contralesional M1 (23, 24) but improve when anodal tDCS is applied to contralesional M1 (21). This may be because the mechanisms mediating the recovery for individuals with severe impairment differ from those of people who are less impaired (4, 25, 26). In people with severe motor deficits, compensatory activation of contralesional M1 may support motor recovery. Similarly, the stage of stroke may also influence the functional role of the contralesional hemisphere in recovery (13).

Prior systematic reviews and meta-analyses have been published on this topic, however with conflicting conclusions, from no indication (27–30) to indication (31, 32) for clinical use. For example, a group of European experts reviewed current evidence on the therapeutic use of tDCS in stroke motor recovery and concluded based on one class I study and one class II study that there was no effect of tDCS using the cathode on contralesional M1 (30). However, meta-analyses that included more studies either found no significant effect (29) or a significant effect (32) but combined studies that administered multiple and single sessions of tDCS in their analyses. In stroke rehabilitation, individuals typically receive multiple sessions of therapy, and thus evaluating the effects of multiple tDCS sessions is relevant to determine whether these effects are cumulative and enhance efficacy. Multiple sessions of tDCS with the cathode on contralesional M1 improved activities of daily living after stroke but did not reduce motor impairment as measured by Fugl–Meyer (FM) upper extremity assessment (31). This latter study (31), along with other meta-analyses (28, 32), also estimated the effect using outcome data gathered at the end of the intervention and thus did not account for baseline characteristics that may influence the amount of behavioral change possible from an intervention.

The purpose of the systematic review and meta-analysis was to determine whether available evidence indicates that cathodal tDCS to contralesional M1 enhances motor performance or recovery post-stroke more than sham tDCS. This work is an update from prior studies (27–29, 32–34) that also report effects of cathodal vs. sham tDCS on motor performance or recovery. Our meta-analysis represents an incremental advance for the following reasons: First, we only included RCTs that involved multiple sessions of tDCS combined with an intervention, which allows us to evaluate the effects of tDCS on motor recovery. Second, we calculated the pooled effect size using a change score, which represents the difference between baseline and follow-up in the outcome measure. This mitigates issues concerning different baseline characteristics that may confound effect estimation when tDCS is only evaluated cross-sectionally across conditions (27). Third, a follow-up assessment time point was used in the calculation of the change score as opposed to an immediate post-tDCS assessment. This allows us to evaluate the effect of cathodal tDCS on the relative permanence of motor recovery. Fourth, we conducted a meta-analysis that focused on the synthesis of the FM upper extremity assessment of impairment. Ongoing discussions have highlighted the need to understand and distinguish between processes related to “true recovery” or restitution of behaviors vs. adaptation and compensation (35–38). Our meta-analysis evaluates the evidence for whether tDCS reduces motor impairment, which may imply that it involves plasticity mechanisms related to neural repair as opposed to the learning of compensatory behaviors. Our study is an advance over prior work that showed an effect of tDCS on measures of body functions/structures and activity levels of the International Classification of Functioning combined (32, 33). It also builds on a prior meta-analysis of *n* = 4 studies that evaluated the effect of multiple sessions of tDCS on the change in FM scores (27).

## Methods

This systematic review and meta-analysis was conducted in accordance with the PRISMA statement and checklist (39).

### Information Sources and Search Strategy

The literature search was performed in collaboration with a medical librarian (HL). We identified studies by systematically searching the following electronic databases, limited from the year 1990: Ovid MEDLINE(R) and Ovid OLDMEDLINE(R) <1946 to October, week 3, 2019; Embase Classic + Embase <1947 to 2019, week 43; and the Cochrane Central Register of Controlled Trials <September 2019 edition>.

We applied a broad search strategy, which we then narrowed down with specific eligibility criteria as outlined in the following “Eligibility Criteria”. We combined the following set of search terms using the Boolean operator “OR”: transcranial direct current stimulation, tDCS, electric stimulation therapy, neuro stimulation, transcranial stimulation, non-invasive brain stimulation, NIBS, direct current stimulation, brain stimulation, cortical stimulation, cranial stimulation. A second set of search terms was also combined using the Boolean operator “OR”: cerebrovascular disorders, intracranial arteriosclerosis, intracranial embolism and thrombosis, stroke, cerebrovascular accident. The Boolean operator “AND” was applied to combine the first two searches, with the limits human and English language. Furthermore, medical sub-headings for each database were used when provided (see Table 1 for the Medline database search strategy and see Supplementary Tables 1, 2 for Embase and Cochrane Central searches, respectively).

**Table 1:** Search strategy: Ovid MEDLINE(R) <1946 to October, week 3, 2019>.

1. Transcranial direct current stimulation/ (2175) 2. (transcranial direct current stimulation or tDCS).mp. (3589) 3. Electric stimulation therapy/ (20014) 4. neuro stimulation.mp. (45) 5. transcranial stimulation.mp. (425)
6. NIBS.mp. (216) 7. noninvasive brain stimulation.mp. (491) 8. direct current stimulation.mp. (3440) 9. brain stimulation.mp. (13844) 10. cortical stimulation.mp. (1974)
11. cranial stimulation.mp. (18) 12. or/1-11 (37154) 13. exp stroke/ (126438) 14. exp cerebrovascular disorders/ (354003) 15. exp intracranial arteriosclerosis/ (10695)
16. exp “intracranial embolism and thrombosis”/ (20812) 17. stroke.mp. (243364) 18. 13 or [17 and (14 or 15 or 16)] (157274) 19. 12 and 18 (1207) 20. limit 19 to (English language and humans and yr = “1990–current”) (1057)

### Eligibility Criteria

The eligibility criteria were specific in order to address the updates represented by this systematic review and meta-analysis in comparison to prior work reviewed in the “INTRODUCTION”. Articles retrieved from the search were included if they met the following criteria: (1) participants: individuals with hemorrhagic or ischemic stroke (no lesion location limit), >18 years of age and >7 days post-stroke (i.e., subacute and chronic stages), with motor impairment to the upper limb; (2) intervention: tDCS applied online or offline, with any motor rehabilitation intervention or motor training paradigm; the cathode electrode is placed on contralesional M1 and the anode electrode on the contralateral supraorbital area; (3) comparison: the condition of sham tDCS; (4) outcomes: change in any clinically validated assessment of physical function, activity, or participation (e.g., Fugl–Meyer, action research arm test, etc.) or change in a movement performance variable (e.g., movement time, accuracy, smoothness); (5) time: one or more sessions of the intervention; and (6) type of publication: articles published in English and peer-reviewed. The included studies were all clinical in nature.

We excluded the following: review articles, conference abstracts, and single case reports.

### Study Selection, Data Extraction, and Quality Assessment

Three investigators (JLC, AS, and CPS) independently screened the titles and abstracts of all articles identified in the search and performed a full-text evaluation of the articles against the inclusion and exclusion criteria to determine eligibility. The following information was extracted for each included paper: participant characteristics, intervention program, tDCS parameters, outcome measures, and sample size. Discrepancies were resolved by consensus between the investigators.

Two investigators (JLC and JDE) independently rated the quality of evidence using the Physiotherapy Evidence Database (PEDro) (40). The PEDro scale includes 11 items, the first of which is not used to calculate the PEDro score. Scores of 9 and 10 reflect studies of excellent quality, 6–8 as good, 4 and 5 as fair, and <4 as poor (41). Cohen's kappa was performed to measure the inter-rater agreement. Discrepancies were resolved by consensus between the two investigators.

### Data Synthesis and Analysis

The extracted data were summarized in a table where differences and similarities were noted. A meta-analysis was conducted using Review Manager (version 5.3). The outcome of interest was the standardized mean difference (SMD) of the “change” score on the FM upper extremity assessment between cathodal tDCS and sham tDCS. Change is defined as the difference between baseline (i.e., before tDCS) and follow-up (i.e., after tDCS). As our goal was to assess long-term changes in motor skill retention, follow-up was defined as any assessment time point that did not occur immediately after tDCS (e.g., same day after the last tDCS session). We also performed a sub-group analysis to determine if the effects of cathodal tDCS differ between people in the chronic (>6 months) vs. sub-acute (7 days to 6 months) stage of stroke. A random-effects model was used to account for differences in effect sizes between studies (42). Ninety-five percent confidence intervals (95% CI) were calculated for the overall effect. Heterogeneity was assessed with chi-square, *I*^2^, and τ^2^ statistics.

## Results

### Search Results

**Figure 1:**
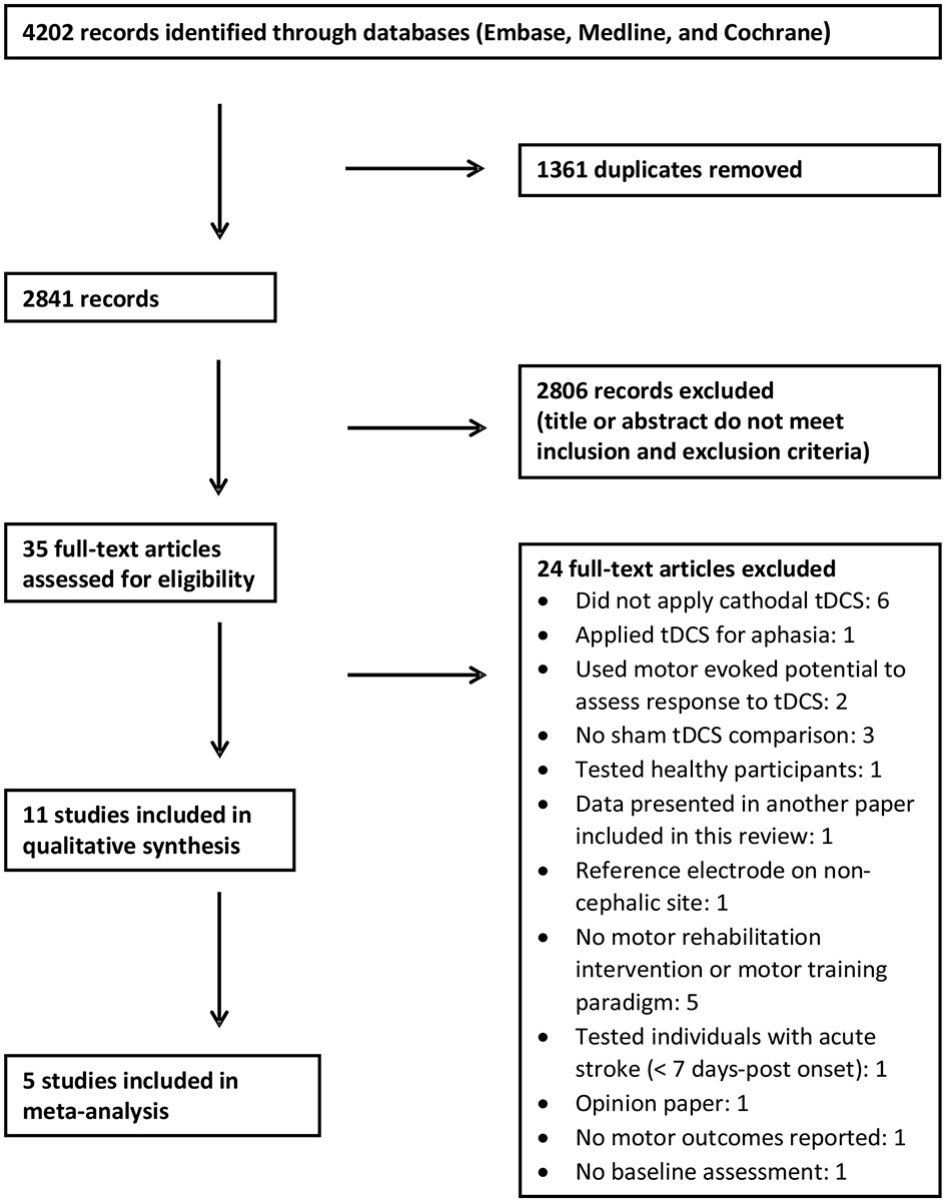
Flow chart depicting the study selection.

We identified 4,202 records using our search criteria (Figure 1). After removal of duplicates, we screened the titles and abstracts of 2,841 articles, 35 of which further underwent a full-text review. Of these, 24 articles were excluded after the full-text review (see Figure 1 for details). Eleven articles met the inclusion and exclusion criteria and were included in the systematic review (43–53); five of these studies were included in the meta-analysis (45, 47, 48, 50, 53).

### Characteristics of Studies

#### Quality

There was “substantial agreement” (54) between the two raters' initial PEDro scores (*κ* = 0.612, *p* < 0.001). Out of 121 ratings (11 items by 11 studies), the two raters disagreed on 14 items. These disagreements were resolved by discussion, and the final results of the PEDro quality assessment are shown in Table 2. The scores ranged from 6 to 10. Six studies are considered excellent in quality (PEDro score 9 and 10), and five studies are considered good in quality (PEDro score 6–8). There were only two PEDro items for which few studies scored points on: (1) concealed allocation (item 3) of participants occurred in five studies and (2) therapist blinding (item 6) occurred in six studies.

**Table 2:** PEDro scores.

**References**	**PEDro item**											
	**1^a^**	**2**	**3**	**4**	**5**	**6**	**7**	**8**	**9**	**10**	**11**	**Total**
Boggio et al. (43)	Yes	1	0	0	1	0	1	1	1	1	1	7
Fleming et al. (44)	Yes	1	0	1	1	0	0	1	0	1	1	6
Fregni et al. (46)	No	1	0	1	1	0	1	1	1	1	1	8
Hesse et al. (45)	Yes	1	1	1	1	1	1	1	1	1	1	10
Khedr et al. (53)	Yes	1	1	1	1	1	1	1	1	1	1	10
Kim et al. (47)	Yes	1	1	1	1	1	1	1	0	1	1	9
Nair et al. (48)	Yes	1	0	1	1	1	1	0	1	1	1	8
Nicolo et al. (49)	Yes	1	1	1	1	0	1	1	1	1	1	9
Rocha et al. (50)	Yes	1	1	1	1	1	1	1	1	1	1	10
Stagg et al. (51)	Yes	1	0	1	1	0	0	0	1	1	1	6
Zimerman et al. (52)	Yes	1	0	1	1	1	1	1	1	1	1	9
Total number of studies		11	5	10	16	6	9	9	9	11	11	

a *Not included in the total score*.

*PEDro items: 1, eligibility criteria specified; 2, participants randomly allocated to groups; 3, allocation concealed; 4, groups similar at baseline; 5, participants were blinded; 6, therapists were blinded; 7, assessors were blinded; 8, data available for more than 85% of participants; 9, participants received the treatment as allocated or intention-to-treat analysis was used; 10, statistical analyses were reported; 11, point measures and variability of data reported; 0, no; 1, yes*.

#### Participants

A total of 289 participants with stroke who met study the inclusion and exclusion criteria were included in the analyses (see Table 3 for information on the participants' characteristics). Across studies, the average age of the participants ranged between 50 and 69.9 years. There were 169 male (64%) and 96 female (36%) participants; one study did not report sex. Information about gender was not reported in any study. One hundred one participants (96%) were reported as right-handed, four participants (4%) were left-handed, and three studies did not report handedness.

**Table 3:** Participants' characteristics.

**References**	**Number of participants**	**Age (mean ± SD years; range)**	**Sex Male/female**	**Handedness Right/Left**	**Time since stroke (mean ± SD, range)**	**Type of stroke Ischemic/ hemorrhagic**	**Lesion hemisphere Right/left**	**Stroke location Cortical/ subcortical/ both**	**Baseline severity (mean ± SD, range)**
**Cross-over designs**									
Boggio et al. (43) Experiment 1	4	60.75 ± 13.15; 44–75	4/0	4/0	34.5 ± 27.74 months; 12–72	-	1/3	0/4/0	MRC: 4.3 ± 0.50; 3.8–4.8
Fleming et al. (44)	24	58.9 ± 12.57; 34–76	-	22/2	20.4 ± 27.79 months; 3–124	20/4	12/12	7/16/1	JTT: 78.8 ± 73.4; 29.7–314.1
Fregni et al. (46)	6	53.67 ± 16.64; 28–75	4/2	6/0	27.08 ± 24.27 months; 12–72	-	3/3	1/3/2	MRC: 4.2 ± 0.37; 3.5–4.5
Stagg et al. (51)	13	66.38 ± 13.08; 30–80	10/3	13/0	40.23 ± 16.16 months; 18–70	12/1	4/9	6/7/0	FM: 43.2 ± 17.2; 16–66
Zimerman et al. (52)	12	58.3 ± 13.9; 31–72	6/6	12/0	33.41 ± 16.49 months; 12–64	12/0	7/5	0/12/0	FM: 63.83 ± 1.27; 61–65
**Randomized controlled trials**									
Hesse et al. (45)	Anode: 32 Cathode: 32 Sham: 32	63.9 ± 10.5; 39–79 64.5 ± 8.6; 46–79 65.6 ± 10.3; 39–79	20/12 18/14 21/11	- - -	3.4 ± 1.8 weeks; - 3.8 ± 1.4 weeks; - 3.8 ± 1.5 weeks; -	32/0 32/0 32/0	14/18 15/17 16/16	25/7/0 24/8/0 26/6/0	FM: 7.8 ± 3.8; - FM: 7.9 ± 3.4; - FM: 8.2± 4.4; -
Khedr et al. (53)	Anode:14 Cathode: 13 Sham: 13	58 ± 9.5; 38–67 60 ± 9.6; 40–75 57 ± 7.5; 44–66	8/6 10/3 8/5	- - -	13.8 ± 5.8 days; 7-25 12.3 ± 4.4 days; 7-20 12.6 ± 4.6 days; 7-22	14/0 13/0 13/0	7/7 8/5 7/6	8/4/2 4/6/3 6/4/3	NIHSS: 10.8 ± 2.0; 7–10 NIHSS: 9.9 ± 1.5; 7–12 NIHSS: 11.3 ± 1.5; 9–13
Kim et al. (47)	Anode: 6 Cathode: 5 Sham: 7	55.3 ± 16.4; - 53.6 ± 14.9; - 62.9 ± 9.2; -	5/1 4/1 4/3	- - -	34 ± 27.1 days; - 19.4 ± 9.3 days; - 22.9 ± 7.5 days; -	6/0 5/0 7/0	3/3 1/4 5/2	1/4/1 2/1/2 2/4/1	FM: 31 ± 11.2; - FM: 39.2 ± 19.0;- FM: 41± 13; -
Nair et al. (48)	Cathode: 7 Sham: 7	61 ± 12; - 56 ±15; -	5/2 4/3	7/0 7/0	33 ± 20 months; - 28 ± 28 months; -	7/0 7/0	4/3 2/5	0/2/5 0/3/4	FM: 30 ± 11; - FM: 31 ± 10; -
Nicolo et al. (49)	Cathode: 14 Sham: 13 cTBS: 14	68.5 ± 10.8; - 64.3 ± 17.1; - 62.4 ± 12.3; -	8/6 8/5 7/7	13/1 13/0 13/1	5.5 ± 1.7 weeks; - 4.7 ± 1.4 weeks; - 5.3 ± 1.8 weeks; -	10/4 10/3 13/1	9/5 10/3 10/4	2/4/8 1/6/6 2/4/8	FM: 18.8 ± 15.5;- FM: 18.6 ± 17.2;- FM: 16.9 ± 13.6;-
Rocha et al. (50)	Anode: 7 Cathode: 7 Sham: 7	58.3; 49–64 58.5; 41–71 58.5; 46–70	6/1 5/2 4/3	7/0 7/0 7/0	27.5 months; 9–48 34.2 months; 10–67 26.5 months; 6–46	- - -	3/4 3/4 4/3	- - -	FM: 44.6 ± 4.1; - FM: 51.6 ± 4.2; - FM: 51 ± 8.9; -

*MRC, Medical Research Council muscle strength test (maximum score 5); FM, Fugl–Meyer upper extremity assessment (maximum score 66); JTT, Jebsen Taylor Test (timed, s); NIHSS, National Institutes of Health Stroke Scale; MRS, Modified Rankin Scale*.

Four studies tested participants in the early sub-acute stage (i.e., 7 days to 3 months post-stroke), one study tested participants spanning the late sub-acute (3 to 6 months) and chronic stages (>6 months post-stroke), and six studies tested participants in the chronic stage (>6 months post-stroke). Two hundred forty-five participants (95%) had an ischemic stroke, and 13 participants (5%) had a hemorrhagic stroke; three studies did not report stroke sub-type. Stroke lesions were in the right hemisphere in 148 participants (51%) and in the left hemisphere in 141 participants (49%). Stroke lesions were cortical in 117 participants (44%), subcortical in 105 participants (39%), and both cortical and subcortical in 46 (17%) participants; one study did not report lesion location. Individuals with mild, moderate, and severe strokes were tested across the studies. Seven studies assessed baseline severity using the FM assessment: one study tested severely affected individuals (average FM scores <10) (45), one study tested people with mild impairment (average FM score >60) (52), and the other studies tested people with scores in between.

Nine studies reported whether the participants experienced adverse events (AE). Of these studies, two reported the presence of AEs, and the participants withdrew because of headache (*n* = 2) (44, 47) and dizziness (*n* = 1) (47). Two studies did not mention whether AEs were experienced (48, 51).

#### Study Interventions

Table 4 presents information on study characteristics and results.

**Table 4:** Study characteristics and results.

**References**	**Number of sessions**	**Training or intervention**	**Transcranial direct current stimulation (tDCS) protocol: When Intensity (mA) Duration (minutes) Electrode size (cm^**2**^)**	**Outcome measures**		**Result (primary outcome)**	**PEDro score**
				**Primary (P) Other (O)**	**When measured**		
**Cross-over designs**							
Boggio et al. (43) (Experiment 1)	4 (1/week) each of anode, cathode, and sham tDCS 2 week washout	Jebsen Taylor Test	Online 1 20 35	P: Jebsen Taylor Test	Baseline, pre, post	(1) ANOVA on change score: effect of stimulation, *p* = 0.009; (2) *post-hoc* comparison cathode vs. sham *p* = 0.016	7
Fleming et al. (44)	1 each of anode, cathode, dual, and sham tDCS 1 week washout	Motor sequence learning task	Online 1 20 25	P: Jebsen Taylor Test	Pre, post	(1) ANOVA on change score: effect of stimulation, *p* = 0.003; (2) *post-hoc* comparison cathode vs. sham *p* = 0.003	6
Fregni et al. (46)	1 each of anode, cathode, and sham tDCS 2 day washout	Jebsen Taylor Test	Online 1 20 35	P: Jebsen Taylor Test	Baseline, pre, post	(1) ANOVA; interaction of stimulation and time, *p* = 0.002; (2) ANOVA for cathode tDCS; main effect of time, *p* = 0.001	8
Stagg et al. (51)	1 each of anode, cathode, and sham tDCS 1 week washout	Response time and grip force tasks	Online 1 20 35	P: response times and grip strength	Pre, post	(1) ANOVA: interaction of stimulation and time, *p* = 0.005; (2) paired *t*-tests on change score: cathode vs. sham *p* = 0.048; pre vs. post cathode *p* = 0.92	6
Zimerman et al. (52)	1 each of cathode and sham tDCS 9 day washout	Motor sequence learning task	Online 1 20 25	P: number of correct sequences O: total number of sequences per block	Post, post 90 min, post 24 h	(1) ANOVA: interaction of stimulation and time, *p* = 0.02; (2) *post-hoc* comparison cathode vs. sham *p* < 0.05	9
**Randomized controlled trials**							
Hesse et al. (45)	30 (5 days/week for 6 weeks)	Robotics therapy	Online 2 20 35	P: Fugl–Meyer O: strength, tone, Barthel Index, box and block	Pre, post, 3 months	ANOVA on change score: effect of time, *p* < 0.001, no effect of group or interaction	10
Khedr et al. (53)	6 consecutive days	In-patient therapy	Offline, followed by therapy 2 25 35	P: NIHSS, Orgogozo scale, Barthel Index, strength	Pre, post, 1, 2, and 3 months	(1) ANOVA: interaction of group (tDCS vs. sham) and time, *p* < 0.005; (2) ANOVA: interaction of group (cathode vs. sham) × time, *p* = 0.017)	10
Kim et al. (47)	10 (5 days/week for 2 weeks)	Conventional therapy	Online 2 20 25	P: Fugl–Meyer, Barthel Index	Pre, post 1 day, 6 months	(1) ANOVA: interaction of group and time, *p* = 0.017; (2) *post-hoc* comparisons final score at 6 months, cathode vs. sham, *p* < 0.05	9
Nair et al. (48)	5 (1 day/week for 1 week)	Occupational therapy	Online 1 30 35	P: Fugl–Meyer, range of motion	Pre, post, post 7 days	ANOVA: interaction of group and time, *p* = 0.048	8
Nicolo et al. (49)	9 (3 days/week for 3 weeks)	Physical therapy	Online 1 25 35	P: composite motor score (Fugl–Meyer, Box and Block, Nine Hole Peg Test, Jamar dynamometer) O: assessments comprising composite score, analyzed separately; brain connectivity	Baseline, pre, post, post 30 days	Kruskal–Wallis test on change scores between post and pre, *p* = 0.61	9
Rocha et al. (50)	12 (3 days/week for 4 weeks)	Constraint-induced movement therapy (CIMT)	Offline, followed by CIMT 1 Anode: 13 min Cathode: 8 min 35	P: Fugl–Meyer O: motor activity log, grip strength	Pre, post, 1 month	(1) ANOVA: interaction of group and time, *p* = 0.035; (2) unpaired *t*-test, cathode vs. sham, *p* > 0.05	10

*Highlighted cells indicate a significant difference between cathode vs. sham tDCS*.

##### Study design

There were five studies that employed a cross-over design to evaluate the effects of different types of tDCS on the same participant (i.e., sham *vs*. cathode and/or anode montages). Four of these studies probed the effects of a single tDCS session, and one study tested the effects of four sessions. The number of participants tested in these studies ranged from four to 24, two studies tested six or less participants, two studies tested 12 to 13 participants, and one study tested 24 participants.

There were six randomized controlled trials (RCTs). Five studies evaluated the effects of five to 12 sessions of tDCS, three studies tested <10 participants per group, and two studies tested 13 to 14 participants per group. One study evaluated the effects of 30 sessions of tDCS on 32 participants per group.

##### Behavioral paradigm

Five studies that employed a cross-over design asked the participants to perform motor tasks ranging from the Jebsen Taylor Hand Function Test, motor (finger) sequence learning, hand movement in response to cues, and grip force. Of the six RCTs, four employed physical or occupational therapy, one implemented robotics, and one implemented constraint-induced movement therapy.

##### tDCS parameters

Stimulation intensity was either 1 mA (eight studies) or 2 mA (three studies). Stimulation duration ranged from 20 min (seven studies), 25 min (two studies), or 30 min (one study). One study applied 8 min of stimulation with the cathodal on M1 and 13 min of stimulation with the anode on M1. The electrode size applied was either 25 cm^2^ (three studies) or 35 cm^2^ (eight studies).

Nine studies applied tDCS online, concurrent with the behavioral paradigm, and two RCTs applied tDCS offline before the therapy.

#### Outcome Measures and Outcomes

Studies that employed a cross-over design either administered a clinical test of motor activity, The Jebsen Taylor Hand Function Test (three studies), or study specific measures of response times and accuracy (two studies). In four studies, the outcomes were assessed before and after the tDCS intervention; only one study tested skill retention 24 h post-intervention. Four studies reported a significant effect for cathodal compared to sham tDCS (43, 44, 46, 52). While one study showed a significant difference between cathodal and sham tDCS in the percent response time change from baseline, there was, in fact, no difference in response times between baseline and post-testing for the cathodal tDCS condition (51).

Five of the included RCTs administered the FM assessment of motor impairment, and one trial evaluated several outcomes including the NIHSS and Barthel Index. All RCTs included a follow-up testing session, which ranged from 7 days to 6 months post-intervention. Three of the six RCTs report a significant effect for cathodal compared to sham tDCS (47, 48, 53).

### Meta-analysis

We conducted a meta-analysis of RCTs that reported the FM assessment of motor impairment as the primary outcome (*n* = 5) and compared the effects of cathodal to sham tDCS over multiple days. In one trial, the sham stimulation group comprised of participants who underwent sham tDCS (*n* = 5) or sham cTBS (*n* = 8) (49). The follow-up time point ranged from 7 days to 6 months. Three trials tested people with chronic stroke, and two tested people in the early sub-acute stage.

**Figure 2:**
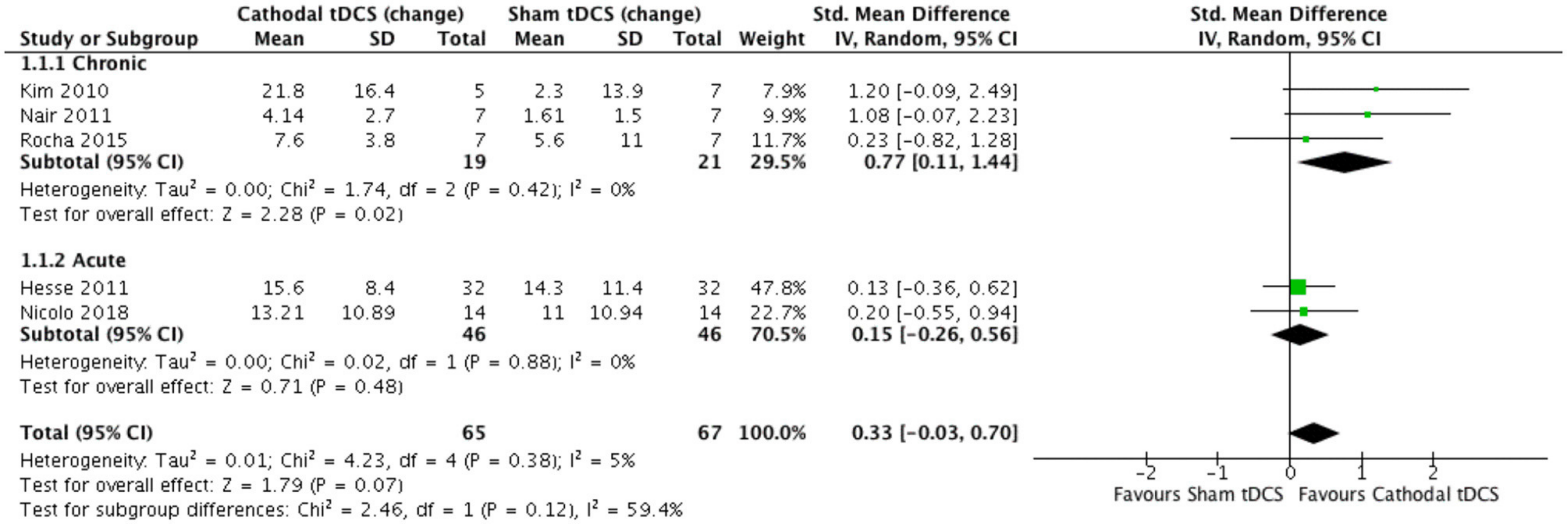
Effect (standardized mean difference) of cathodal transcranial direct current stimulation (tDCS) *vs*. sham tDCS on the change in Fugl–Meyer upper extremity score.

The test for subgroup differences indicated no statistically significant subgroup effect (*p* = 0.12), suggesting that stage of stroke did not modify the effect of cathodal tDCS in comparison to sham tDCS. The pooled effect estimate favored cathodal tDCS in participants with chronic stroke: cathodal tDCS (*n* = 19) significantly reduced motor impairment compared to sham tDCS (*n* = 21) (SMD = 0.77, 95% CI = 0.11 to 1.44, *p* = 0.02, Figure 2). There was evidence for low heterogeneity (χ^2^ = 1.74, *p* = 0.42; *I*^2^ = 0%; τ^2^ = 0.00) across trials. In people in the early sub-acute stroke stage, the effect of cathodal tDCS (*n* = 46) was not significantly different from the effect of sham tDCS (*n* = 46) (SMD = 0.15, 95% CI = −0.26 to 0.56, *p* = 0.48, Figure 2). There was evidence for low heterogeneity (χ^2^ = 0.02, *p* = 0.88; *I*^2^ = 0%; τ^2^ = 0.00) across trials.

Combining data from both chronic and early sub-acute stages of stroke, cathodal tDCS (*n* = 65) did not significantly reduce motor impairment compared to sham tDCS (*n* = 67) (SMD = 0.33, 95% CI = −0.03 to 0.70, *p* = 0.07, Figure 2). There was evidence for low heterogeneity (χ^2^ = 4.23, *p* = 0.38; *I*^2^ = 5%; τ^2^ = 0.01) across trials. Bias was not formally tested given that there were fewer than 10 studies in the meta-analysis (55).

## Discussion

The aim of this systematic review and meta-analysis was to evaluate the evidence for an effect of cathodal tDCS to contralesional M1 relative to sham tDCS in people with stroke motor impairment. The studies evaluated in this systematic review were considered good to excellent in quality. We pooled data from five RCTs to test the effects of multiple tDCS sessions on upper limb motor impairment as measured by the FM assessment. The effect of cathodal tDCS to contralesional M1, relative to sham tDCS, was small to medium (SMD = 0.33) and did not significantly reduce motor impairment (*p* = 0.07). The heterogeneity across studies was low, and the effect was also not different between people in the early sub-acute vs. chronic phase of stroke. We conclude that, while the effects were small, they were consistent and thus could be clinically meaningful in certain sub-populations. Studies with larger sample sizes selected based on biomarkers may help identify responders from non-responders.

The findings from our meta-analysis are comparable to the effects previously reported that also evaluated the change score. Marquez *et al*. reported an SMD of 0.39, *p* = 0.08; they pooled seven studies in their meta-analysis, three of which tested the effects of multiple tDCS sessions (29). Chhatbar et al. reported an SMD of 0.43, *p* = 0.2, pooling data from four studies testing multiple tDCS sessions (27). Three studies (45, 47, 48) were common across these meta-analyses, and we included an additional two published more recently (49, 50). Taken together, there is modest evidence to support the use of cathodal tDCS to contralesional M1 in reducing motor impairment. To better understand the nature of these improvements at the impairment level, future studies could report changes for each sub-section of the FM upper extremity assessment and/or obtain kinematic measures that enable the precise quantification of movement patterns (38, 56).

We now consider factors that may explain the small-to-medium effect sizes observed with tDCS, which may help us to better target future research studies in this area. First, the notion of an impaired IHI that hinders motor recovery may not apply to all stroke survivors (17). A one-size-fits-all approach of cathodal tDCS to contralesional M1 may lead to a small and non-significant effect since not every individual can benefit. Supporting evidence comes from studies included in our systematic review and meta-analysis. Zimmerman et al. tested well-recovered individuals and found enhanced motor sequence performance that was maintained 24 h after practice (52). In contrast, two RCTs that tested individuals with severe motor impairment (45, 49) showed no difference in motor outcomes between people who received cathodal vs. sham tDCS. RCTs that demonstrated significant findings with large effect sizes tested people who were less impaired (47, 48). Thus, while the findings from our meta-analysis were not significant (*p* = 0.07), it may be that future studies with larger samples and a set of stratification variables to derive sub-groups are required. Refined participant selection criteria may identify individuals with the greatest capacity to benefit for this intervention (57). Our ongoing work (ClinicalTrials.gov ID: NCT02473549) evaluates whether the amount of damage to the corticospinal tract predicts response to tDCS on contralesional M1. Participant selection based on validated biomarkers may also allow for a way to reduce variance and increase statistical power (58).

A second reason for a small-to-medium effect size may be that the dose of tDCS applied is insufficient. Many studies, including those in this systematic review and meta-analysis, typically employ 1-mA current for 20 min applied over an electrode area of 35 cm^2^. There is a positive dose–response relationship whereby the application of a higher current density is associated with greater motor recovery (27). Higher current density is also associated with less variability in corticospinal excitability (59). Therefore, higher current densities may be required to sufficiently engage the targeted area for each individual. This may be especially relevant given the inter-individual variability related to, for example, anatomy and lesion characteristics (60). To control for this variability, computational modeling of current flow through the brain may also permit researchers to individualize dose and spatial targeting (61).

One unanticipated observation was that some studies did not directly test whether the effects of tDCS are long lasting. This is important to incorporate for future research because we are concerned if the skills practiced lead to permanent improvements (62) as evaluated by testing motor performance after some delay from the practice period. All RCTs did evaluate motor performance at a delayed follow-up period. However, of the five studies using a cross-over design, only one study evaluated retention, tested 24 h later, and showed that performance was better for the cathodal vs. sham tDCS groups (52). Three studies report immediate improvement on motor performance after cathodal relative to sham tDCS; however, since retention was not tested, one cannot infer if these improvements would have been maintained.

Future research may also need to consider the psychometric properties of assessments used to evaluate improvement from tDCS. The FM upper extremity assessment has excellent psychometric properties (63), is commonly used in clinical practice, and is highly recommended for research practice (38). The assessment demonstrates longitudinal stability in terms of item difficulty order and is thus valid for measuring change in motor state over time (64). However, it has floor and ceiling effects (63), and items are not linearly related as a gain of points at the bottom vs. the top end has a different meaning (65). Therefore, future studies that use the FM should take these factors into account. Some propose using a sliding dichotomous outcome or responder analysis, whereby subgroups are specified before the trial, and response to therapy is defined differently for each subgroup (65). Another approach is to implement a Rasch-rescaled version of the assessment (66).

Another noteworthy observation was that more males (64%) than females (36%) participated in the research studies, which limits the generalizability of findings. Future work should consider sex as a variable of interest to determine if there are biological differences in response to tDCS for stroke motor recovery. To better understand this potential bias, one can also explore social–pragmatic barriers to research participation (67) that may differentially affect females. Otherwise, the characteristics of the participants studied in this review are mostly consistent with those of the general population of people with stroke. The average age ranged from 50 to 70 years, with most participants being right-hand dominant (95%), having had an ischemic stroke (94%), with equal representation of right (49%) or left (51%) hemispheres affected, and with cortical (42%) vs. subcortical (42%) lesions.

The stage of stroke recovery may be an important moderator variable to further explore in future research that targets tDCS to contralesional M1. Corticospinal and intracortical excitability changes occur as a function of time since stroke, which may reflect how motor output is generated (68). Few studies have evaluated excitability changes longitudinally in the same individuals. One study found that cortical M1 excitability in the lesioned hemisphere correlates with assessments of motor activity in the acute stage, but only weakly at 3 and 6 months post-stroke (69). In contrast, intracortical M1 excitability in the unaffected hemisphere correlated strongly with assessments of motor activity at 3 months but not in the acute stage (69). These findings align with those from another longitudinal study showing a time-dependent role of the contralesional M1: activity in contralesional M1 negatively affects motor performance during the early sub-acute phase (1 and 2 weeks) but not in the late sub-acute phase (>3 months) (13). A prior meta-analysis also found that cathodal tDCS improved motor outcomes in people with chronic but not acute or sub-acute stroke (32). However, they evaluated data at the post-test/intervention time point and combined findings from cross-sectional studies and RCTs. Our sub-group analysis suggests that the effect does not differ between individuals in the sub-acute vs. chronic stage of stroke. A caveat with our sub-analysis is that a small number of trials and participants contributed data such that the analysis may not be able to detect subgroup differences.

One major limitation of prior work included in this systematic review with meta-analysis is that many studies tested a small number of participants. Of the five studies that employed a cross-over design, four studies tested 13 or less participants. Similarly, five out of six RCTs tested 14 participants or less per group. Studies in the meta-analysis that reported a significant effect of cathodal vs. sham tDCS tested seven participants or less per group (47, 48), and thus this evidence may be unreliable (70). Based on our findings where there may be a small-to-medium effect in the direction favoring cathodal tDCS, to detect an effect size of 0.33 (*α* = 0.05, *β* = 0.80, one-sided independent *t*-test), a sample size of 115 participants per group is required. This supports recommendations for large RCTs in this field (2). The sample size could also be reduced with better selection of participants using biomarkers (58).

## Conclusions

Cathodal tDCS to contralesional M1 did not significantly (*p* = 0.07) improve motor outcomes relative to sham tDCS. The effect size is small to medium (SMD = 0.33), consistent as very little heterogeneity was observed, and aligns with values reported for other meta-analyses using similar data (27, 29). Taken together, cathodal tDCS to contralesional M1 could still be of clinical significance for subpopulations to be identified in future research with larger samples. To maximally harness the effects of tDCS, future research may need to (1) personalize tDCS using evidence-based rationale for how it may facilitate motor recovery from a neurophysiological perspective and (2) be statistically powered to detect an effect.

## Data Availability Statement

The original contributions presented in the study are included in the article/Supplementary Material, further inquiries can be directed to the corresponding author.

## Author Contributions

JC, WS, AT, and JE designed the study. JC, AS, CS, and HL designed and performed the search. JC, AS, and CS performed data collection. JC, CS, and JE performed data analysis. JC wrote the manuscript. JC, AS, CS, HL, WS, AT, and JE edited the manuscript. All authors contributed to the article and approved the submitted version.

## Conflict of Interest

The authors declare that the research was conducted in the absence of any commercial or financial relationships that could be construed as a potential conflict of interest.
