# Supplementary material for: Does Cathodal vs. Sham Transcranial Direct Current Stimulation Over Contralesional Motor Cortex Enhance Upper Limb Motor Recovery Post-stroke? A Systematic Review and Meta-analysis
Source: Front Neurol. 2021 Apr 15;12:626021. doi: 10.3389/fneur.2021.626021 (PMC8083132; doi:10.3389/fneur.2021.626021)
Supplement: Supplementary file 1 [file Table_1.DOCX]

**Supplementary Tables**

**Supplementary Table 1**

**Search Strategy: Embase Classic+Embase <1947 to 2019 Week 43>**

1 transcranial direct current stimulation/ (6308)

2 (transcranial direct current stimulation or tDCS).mp. (7748)

3 electrostimulation therapy/ (11724)

4 neuro stimulation.mp. (151)

5 transcranial stimulation.mp. (744)

6 NIBS.mp. (530)

7 noninvasive brain stimulation.mp. (878)

8 direct current stimulation.mp. (7280)

9 brain stimulation.mp. (25077)

10 cortical stimulation.mp. (3376)

11 cranial stimulation.mp. (43)

12 or/1-11 (45793)

13 exp cerebrovascular accident/ (203090)

14 stroke.mp. (432522)

15 or/13-14 (489396)

16 12 and 15 (2594)

17 limit 16 to (human and english language and yr="1990 -Current") (2217)

**Supplementary Table 2**

**Search Strategy: Cochrane Central Register of Controlled Trials <** **September 2019>**

1 Transcranial Direct Current Stimulation/ (500)

2 (transcranial direct current stimulation or tDCS).mp. (3377)

3 Electric Stimulation Therapy/ or electrostimulation.mp. (3356)

4 neuro stimulation.mp. (18)

5 transcranial stimulation.mp. (133)

6 NIBS.mp. (58)

7 noninvasive brain stimulation.mp. (211)

8 direct current stimulation.mp. (3158)

9 brain stimulation.mp. (2273)

10 cortical stimulation.mp. (181)

11 cranial stimulation.mp. (6)

12 or/1-11 (8371)

13 exp Stroke/ or stroke.mp. or cerebrovascular accident.mp. (55042)

14 exp Cerebrovascular Disorders/ (13287)

15 exp Intracranial Arteriosclerosis/ (412)

16 exp "intracranial embolism and thrombosis"/ (289)

17 or/13-16 (58553)

18 12 and 17 (1465)

19 limit 18 to (english language and yr="1990 -Current") (928)
